# Complexity in Non-Pharmacological Caregiving Activities at the End of Life: An International Qualitative Study

**DOI:** 10.1371/journal.pmed.1001173

**Published:** 2012-02-14

**Authors:** Olav Lindqvist, Carol Tishelman, Carina Lundh Hagelin, Jean B. Clark, Maria L. Daud, Andrew Dickman, Franzisca Domeisen Benedetti, Maren Galushko, Urska Lunder, Gunilla Lundquist, Guido Miccinesi, Sylvia B. Sauter, Carl Johan Fürst, Birgit H. Rasmussen, on behalf of OPCARE9

**Affiliations:** 1 Research and Development Unit in Palliative Care, Stockholms Sjukhem Foundation, Stockholm, Sweden; 2 Department of Learning, Informatics, Management and Ethics, Medical Management Centre, Karolinska Institutet, Stockholm, Sweden; 3 Department of Nursing, Umeå University, Umeå, Sweden; 4 Faculty of Health Sciences, La Trobe University, Melbourne, Australia; 5 Sophiahemmet University College, Stockholm, Sweden; 6 Education and Research Unit, Arohanui Hospice, Palmerston North, New Zealand; 7 Pallium Latinoamérica, Buenos Aires, Argentina; 8 Marie Curie Palliative Care Institute Liverpool, University of Liverpool, Liverpool, United Kingdom; 9 Center for Palliative Care, Cantonal Hospital St. Gallen, St. Gallen, Switzerland; 10 Department of Palliative Medicine, University Hospital Cologne, Cologne, Germany; 11 University Clinic for Respiratory and Allergic Diseases Golnik, Golnik, Slovenia; 12 Department of Radiation Sciences—Oncology, Umeå University, Umeå, Sweden; 13 Palliative Team Västerbergslagen, County Council of Dalarna, Ludvika, Sweden; 14 Clinical and Descriptive Epidemiology Unit, Cancer Prevention and Research Institute, Istituto per lo Studio e la Prevenzione Oncologica, Florence, Italy; 15 Department of Oncology-Pathology, Karolinska Institutet, Stockholm, Sweden; Hospice Africa, Uganda

## Abstract

**Background:**

In late-stage palliative cancer care, relief of distress and optimized well-being become primary treatment goals. Great strides have been made in improving and researching pharmacological treatments for symptom relief; however, little systematic knowledge exists about the range of non-pharmacological caregiving activities (NPCAs) staff use in the last days of a patient's life.

**Methods and Findings:**

Within a European Commission Seventh Framework Programme project to optimize research and clinical care in the last days of life for patients with cancer, OPCARE9, we used a free-listing technique to identify the variety of NPCAs performed in the last days of life. Palliative care staff at 16 units in nine countries listed in detail NPCAs they performed over several weeks. In total, 914 statements were analyzed in relation to (a) the character of the statement and (b) the recipient of the NPCA. A substantial portion of NPCAs addressed bodily care and contact with patients and family members, with refraining from bodily care also described as a purposeful caregiving activity. Several forms for communication were described; information and advice was at one end of a continuum, and communicating through nonverbal presence and bodily contact at the other. Rituals surrounding death and dying included not only spiritual/religious issues, but also more subtle existential, legal, and professional rituals. An unexpected and hitherto under-researched area of focus was on creating an aesthetic, safe, and pleasing environment, both at home and in institutional care settings.

**Conclusions:**

Based on these data, we argue that palliative care in the last days of life is multifaceted, with physical, psychological, social, spiritual, and existential care interwoven in caregiving activities. Providing for fundamental human needs close to death appears complex and sophisticated; it is necessary to better distinguish nuances in such caregiving to acknowledge, respect, and further develop end-of-life care.

*Please see later in the article for the Editors' Summary*

## Abstract 2

In a qualitative study reported by Olav Lindqvist and colleagues, the range of nonpharmacological caregiving activities used in the last days of a patient's life are described.

## Editors' Summary

**Background:**

End-of-life care is a major public health issue, yet despite the inevitability of death, issues related to death and dying are often taboo, and, if mentioned, are often referred to as “palliative care.” There are detailed definitions of palliative care, but in essence, the purpose of palliative care is to relieve any suffering in patients who are dying from progressive illness and to provide the best possible quality of life for both the patient and his or her family. In order to achieve this aim, both pharmacological and non-pharmacological management is necessary, with the latter taking a central role. Recently, a European Commission Seventh Framework Programme project, OPCARE9, aimed to improve the care of dying patients in Europe and beyond by optimizing research and clinical care for patients with cancer in the last days of their life, especially regarding well-being and comfort as death becomes imminent.

**Why Was This Study Done?:**

There is now a growing literature base in non-pharmacological management at the end of an individual's life, particularly in relation to psychological, ethical, and communication issues as well as family-focused and culturally appropriate care. Despite this progress, there is currently little systematic knowledge in how health workers use such non-pharmacological approaches in their efforts to maximize well-being and comfort in patients experiencing their very last days of life. Therefore, in order to advance knowledge in this important clinical area, in this study the researchers reviewed and identified the variety of non-pharmacological caregiving activities performed by different professionals in the last days and hours of life for patients with cancer (and their families) in palliative care settings in the countries that participated in OPCARE9.

**What Did the Researchers Do and Find?:**

The researchers modified an anthropological approach to collect relevant information in participating European countries—Germany, Italy, the Netherlands, Slovenia, Sweden, Switzerland, and the UK—and Argentina and New Zealand. Staff in palliative care settings generated a list of non-pharmacological caregiving activities after discussion about which interventions and activities they carried out with patients and families during the last days of life. This preliminary list of statements was added to if staff performed a new activity when in contact with patients or the patients' family during the last days of life. The researchers then used computer-assisted qualitative data analysis to code the statements.

Using this methodology, the researchers analyzed 914 statements of caregiving activities from 16 different facilities in nine countries. The greatest number of activities described some type of caregiving for an individual carried out through contact with his or her body, such as attending to diverse bodily needs (such as cleaning and moisturizing) while maintaining comfort and dignity. Listening, talking with, and understanding (particularly between professionals and the family) was the next most frequent activity, followed by creating an esthetical, safe, and pleasing environment for the dying person and his or her family, and necessary “backstage” activities, such as organizing paperwork or care plans. Other common activities included observing and assessing, which were often described as being carried out simultaneously with other interventions; just being present (described as increasingly important close to death); performing rituals surrounding death and dying (usually directed to families); guiding and facilitating (encompassing support in a compassionate manner); and finally, giving oral and written information and advice (usually to families).

**What Do These Findings Mean?:**

These findings show that providing for fundamental human needs close to death is complex and sophisticated but ultimately integrated into a common theme of caregiving. This study also identifies a number of areas needing further investigation, such as enhancing the sensory and general environment for the patient and family. Finally, this study suggests that developing a greater level of detail, such as improved terminology for end-of-life care, would enhance appreciation of the nuances and complexity present in non-pharmacological care provision during the last days of life, with potential benefit for clinical practice, teaching, and research.

**Additional Information:**

Please access these websites via the online version of this summary at http://dx.doi.org/10.1371/journal.pmed.1001173.

- The OPCARE9 website details more information about this end-of-life care initiative
- The World Health Organization website defines palliative care, and Wikipedia gives more information (note that Wikipedia is a free online encyclopedia that anyone can edit; available in several languages)
- NHS Choices also provides information about end-of-life care

## Introduction

End-of-life care is a major public health issue in that everyone is affected, by our own mortality and through experiencing the deaths of others close to us. Despite this, issues related to death and dying are often taboo, with nonprofessionals generally ill-prepared to advocate for quality care at the end of life. In palliative cancer care, it has been shown that as death becomes imminent, most active oncologic treatments are no longer physiologically feasible [1], and relief of distress and optimized well-being and comfort instead become primary treatment goals [2],[3]. Both pharmacological and non-pharmacological forms of care may represent approaches for reaching these goals in the last days of life.

Issues of well-being and comfort as death becomes imminent were central to OPCARE9, a recently completed EU Seventh Framework Programme project with the aim of optimizing research and clinical care for patients with cancer in the last days of life. The participating European countries were Germany, Italy, the Netherlands, Slovenia, Sweden, Switzerland, and the UK, with Argentina and New Zealand adding further international representation. A major goal of OPCARE9 was to systematize existing knowledge and identify knowledge gaps.

There appears to be relative consensus within specialized palliative care (PC) that well-being and comfort demand focus on communication and psychological, social, spiritual, and existential care in addition to measures to promote the physical comfort of patients. This is in line with the seminal, now classic work by Cecily Saunders on addressing “total pain” [4],[5], as well as the more recent World Health Organization definition of PC [6]. In specialized PC, great strides have been made in recent decades with respect to improving and researching pharmacological treatments focused on symptom management and relief to improve well-being (see, e.g., [2]). There is also an increasingly robust literature addressing non-pharmacological treatment of psychological, ethical, and communication issues as well as family-focused and culturally appropriate care (see, e.g., [7]–[9]). Despite this rapid expansion of the knowledge base underlying PC provision and the development of guidelines (e.g., [10],[11]), surprisingly little attention has still been paid to how staff use non-pharmacological approaches in their efforts to optimize well-being and comfort in the very last days of life [12]–[15].

One OPCARE9 work package focused on pharmacological and non-pharmacological alleviation of suffering and promotion of well-being and comfort in the last days of life. When beginning to address these issues, we noted that despite our combined long experience in research, policy issues, and clinical practice in PC in general, and end-of-life care in particular, we had little systematic knowledge of the range of non-pharmacological interventions used to promote well-being and comfort for dying people and their families. The clinical complexity of non-pharmacological caregiving—so central to care in the last days of life—remains relatively uninvestigated. The present study, performed in all OPCARE9 countries, therefore aimed to identify the variety of non-pharmacological caregiving activities (NPCAs) carried out by different professionals in the last days and hours of life for patients with cancer and their families in specialized PC settings (we use the term “family” in its broadest sense, to include all significant others). Based on these data, we argue that PC for dying patients and their families in the last days of life is multifaceted and complex, with physical, psychological, social, spiritual, and existential care interwoven in caregiving activities. While this is in accordance with guidelines and recommendations for optimal PC, we complement the existing literature by providing detailed empirical data and analysis of how staff report working to achieve these generally accepted goals in the last days and hours of life.

## Methods

This project was coordinated by the Swedish national OPCARE9 core group, supplemented by one contact person from each national team. Based on previous work [16], we used a variation of a free-listing approach [17],[18] for data collection, as described below. This method is derived from anthropology, to allow identification of relevant issues uncolored by researchers' assumptions.

### Ethics Statement

Ethical principles for research have been followed in accordance with norms in each of the nine involved countries; whether formal ethical review was necessary or not varied by country. All staff contributing data were aware of the purpose of the study and agreed to contribute.

### Data Generation

The team initially trialed the data collection protocol with PC staff in Sweden. One hospice unit and one PC home care unit were asked to brainstorm about which interventions and activities they carried out with patients and families during the last days of life. The brainstorming discussions generated a preliminary list of NPCAs described in detailed, often composite statements. These lists were then positioned in a central place in each unit. Staff members were requested to add to the lists each time they had been in contact with patients/family during the last days of a person's life, and to complement these lists with new NPCAs for up to 4 wk.

Each country representative was then asked to use this approach in at least one specialized PC setting in their home country. The chosen setting could be an inpatient PC or hospice unit, a home care unit, or a setting within the mandate of a PC consultant team. The above strategy was modified with the request that each NPCA conducted be listed only once per patient, in line with the aim of understanding variation rather than frequency of occurrence. We also asked that the category of staff carrying out the NPCA be noted. The country representative was asked to send data in the language in which it was collected, and translated to English. The few statements that were unclear in English translation were checked with the country representative prior to analysis.

### Data Analysis

**Figure 1:**
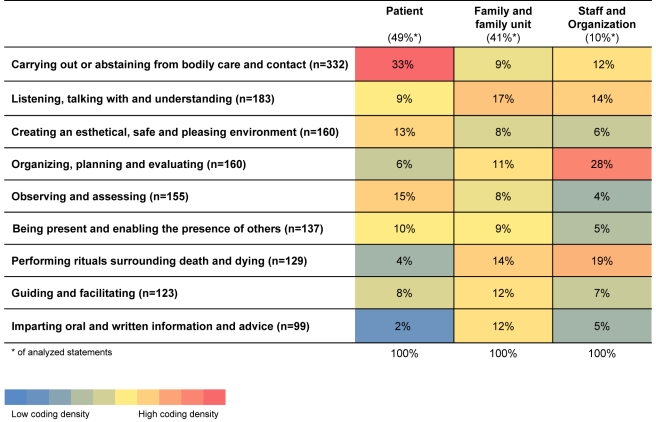
Matrix overview of relative frequencies of codes in each category, by character of activity and its recipient.

The English language statements were compiled in unedited form and entered into NVivo 8, a computer-assisted qualitative data analysis software, for further structuring by a core group of three researchers, O. L., C. T., and B. H. R., working together in different constellations of pairs. We inductively developed a matrix, presented in Figure 1 and Table 1, to describe and explain the complex composite nature of the recorded statements. One coding dimension in the matrix consists of nine categories clarifying the character of the described NPCAs. A second coding dimension describes the recipient or party involved in the NPCA using the following three categories: activities directed toward the dying person alone; activities directed toward or involving the family unit, with or without the involvement of the dying person; and activities directed toward or involving health care staff and care organization, including intra- and inter-professional communication. The analysis group then jointly coded and discussed all statements, to define and distinguish between the categories of NPCAs in each statement. Statements containing more than one NPCA were categorized for each activity, and could be coded into more than one category when applicable.

**Table 1:** Matrix of categories, with examples of coded statements.

Category	Targeted Recipient of the NPCA		
	Patient	Family and Family Unit	Staff and Organization
**Carrying out or abstaining from bodily care and contact**	More physical contact with the patient (take his hand, touch him). Make him feel he is not alone (physician)	Stay with the relatives; give them some comfort, bringing tea for them, bringing comfortable chair for them (volunteer)	Feels good to be able to have this kind of ending, without doing anything special, keeping my fingers out of it, not treating (physician)
**Listening, talking with, and understanding**	Explore the patient's wish, about somebody's presence in particular (physician)	Talked with wife and two friends around bedside of non-responsive patient encouraging stories about him and their life together (social worker)	Find out how staff in community homecare experience the situation by talking to them…. Important that they feel secure and have experience/competence so that they can in turn communicate that to patient and family.… Offer to meet, that they can call us, etc. (nurse)
**Creating an aesthetic, safe, and pleasing environment**	We are trying to give him everything he wants. From the special incense on his table, special drops in his water, his own pillow and slippers beside his bed, even if he is not able to walk (nurse technician)	On two occasions—dying patient wheeled out late afternoon to feel the sunset. Family in attendance. Both families most appreciative. Pictures were taken (nurse)	Difficult to not do anything, to leave—for example when family thought the patient looked nice but I thought it was horrible—hair standing up, dirty shirt on crookedly, the bed in chaos. The values one has collide with those of the family—I thought I'd done a bad job (nurse)
**Organizing, planning, and evaluating**	Check on needs such as orthopedic bed, oxygen tube, etc. (nurse)	Asked the family if there was anything we could have done differently (nurse)	Organize volunteer of hospice service: telephone contact with coordinator (nurse)
**Observing and assessing**	Assess gestures or signs of pain (nurse)	Regularly checked patient and family to judge the comfort of the patient and how the family was doing (nurse)	Assess bereavement within the team (team counselor)
**Being present and enabling the presence of others**	Denies any discomfort. Likes somebody in his room. I sit for awhile and stay silent, holding his hand (physician)	Allow the entrance of the patient's children to the ward to say goodbye (psychologist)	Call the priest (physician)
**Performing rituals surrounding death and dying**	I stay in the room and pray for the patient (nurse)	Changing behavior when the patient is dying, knocking on the outside door instead of ringing the doorbell when the patient is dying (nurse)	Ritual: the whole multi-professional team has the opportunity to take leave of a patient (whole team)
**Guiding and facilitating**	Confirm for the patient that he is in his last days of life (legitimate sense of dying) (psychologist)	Give support in conflicting feelings like: on one hand, not wanting to miss patient, on the other hand, thinking it will be better if death occurs (nurse)	Call the team to give support and comment on news (nurse)
**Imparting oral and written information and advice**	Even if the patient is sleepy, speak to him and explain what you are doing (physician)	At the start of shift called daughter to inform her about the deterioration of mother (nurse)	Tell the doctor on call that the patient is in the last days of life (physician)

The type of staff making the statement is given in parentheses after each statement.

The vast majority of the statements were composites, consisting of more than one NPCA, and have therefore generated multiple codes. This can be exemplified by the statement: “[Mister H] not responding. Checking the saturation of oxygen. Mister H is not showing any discomfort. Dressing changed. Urine the color of amber in Foley. Covers aligned. Some words for relaxation and consolation given.”

Different portions of this statement have been coded in the first dimension under the categories: “observing and assessing” (“not responding”; “checking the saturation of oxygen”; “Mister H is not showing any discomfort”; “urine the color of amber in Foley”); “carrying out or abstaining from bodily care and contact” (“dressing changed”); “creating an aesthetic, safe, and pleasing environment” (“covers aligned”); and “listening, talking with, and understanding” (“some words for relaxation and consolation given”). In the second dimension, this statement has been coded as only related to the dying person.

A final perusal of the coded material was conducted by the core group together, to check the data, compare and differentiate the final categories, and ensure their consistency. These categories were presented to and discussed by the country representatives (co-authors) at an OPCARE9 meeting, as well as in writing.

## Results

The free-listing exercise generated reports of 985 statements of caregiving activities from 16 different facilities (ten hospices/PC units, three palliative home care teams, and three consultant teams) in nine countries, with 914 statements underlying this analysis. Twenty-two statements were omitted from analysis because they either related only to pharmacological rather than non-pharmacological care or were incomplete or incomprehensible statements. Forty-nine statements were duplicates and omitted from final analysis. Statements were deemed duplicate when they were verbatim repetitions, generated from the same country and staff group.

Approximately 80% of the statements came from registered nurses or other nursing staff, with another 15% reported by physicians. The remainder consisted of between one and 20 statements from at least one representative of the following staff groups: day care coordinators, deacons or deaconesses, occupational therapists and physiotherapists, priests, psychologists, social workers, spiritual counselors, team counselors, and volunteers.

Although the categories of activities are not mutually exclusive, in the presentation below we describe them separately for the sake of clarity. Figure 1 presents the coding matrix, showing both dimensions, i.e., the NPCA and the recipient of the NPCA, indicating the relative distribution of the codes (coding density). Table 1 presents the coding matrix, with illustrative examples.

### Carrying Out or Abstaining from Bodily Care and Contact

The greatest number of NPCAs in the statements described some type of caregiving for an individual carried out through contact with his/her body, be it the dying person's or family member's body. Even statements in which staff reflected upon their role in providing physical care are included here, as exemplified in Table 1. Such reflections also exemplify how abstaining from bodily care is expressed as a purposeful activity in its own right. NPCAs describing care through a dying person's body include those about attending to diverse bodily needs while maintaining comfort, dignity, and a connection with the individual's daily life, e.g., by cleaning and protecting the skin using the person's favorite ointments. Caregiving was often said to be carried out in interaction with the dying person: “When it comes to a certain kind of wounds one would like to act quickly because of the smell, but if you listen to the patients, they tell us how to manage that.”

**Figure 2:**
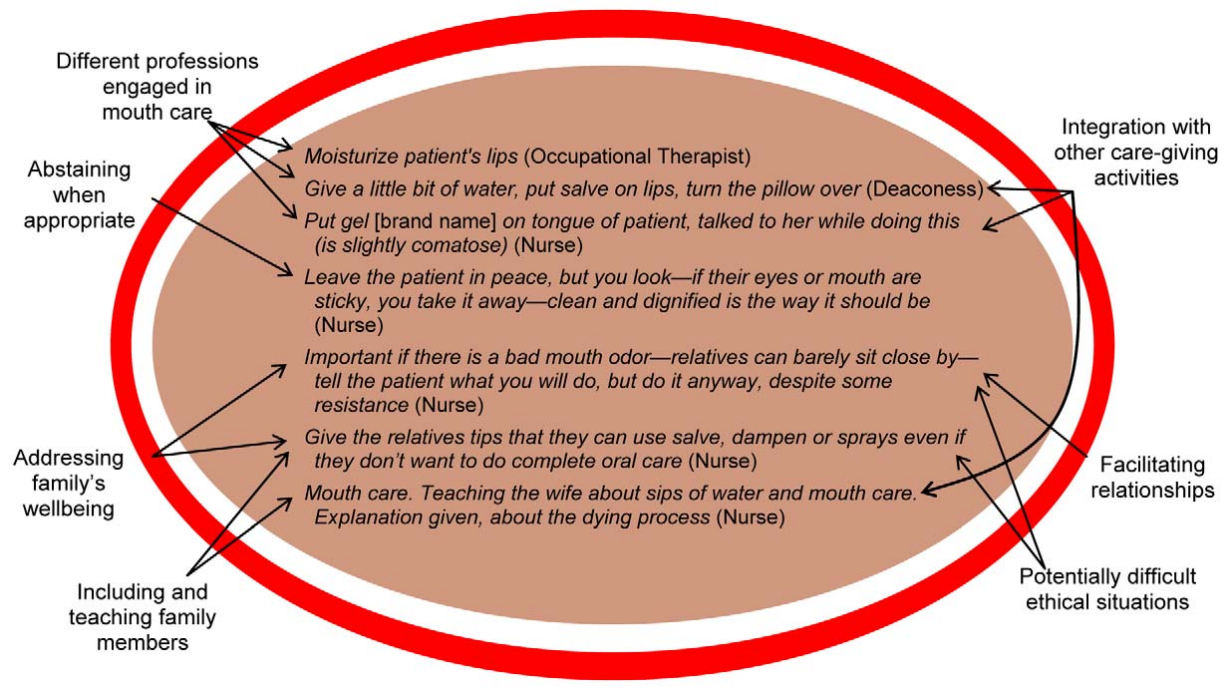
Examples of statements related to mouth care from the category “carrying out or abstaining from bodily care and contact” (total *n* = 54).

Mouth care was particularly prominent, consisting of a wide variety of different activities. These ranged from generic statements to detailed descriptions of different ways to clean or moisten a person's mouth, lips, and tongue, to teaching family members to provide oral care for the individual's comfort. This variation in what may at first be considered a basic and trivial form of care is illustrated in Figure 2. It becomes evident that different professionals both engage in mouth care, and also consider when to abstain from it; that mouth care is integrated with other caregiving activities; that it is a form of facilitating relationships in which even family members' well-being is taken into consideration; and that potential ethical issues are addressed through this form of care.

Other NPCAs that were notably common in these data include positioning the dying person using pillows in creative manners to maximize comfort. The needs and comfort of family members were also addressed in numerous activities (see Table 1 for examples).

Even measures aimed at “comforting,” rather than only providing bodily comfort, are included here. In such activities, contact with an individual's body mediates what often otherwise might be deemed as within the psychosocial realm, e.g., holding a person's hand, light touch, and/or stroking. Efforts at comforting through the body are often clarified by the use of expressions like “carefully turning” and “cut up the sweater in the back, to ease change of clothes,” explaining an intention to be considerate and not purely instrumental in providing care. Such consideration could also be expressed more implicitly, e.g., “smooth out the sheets, get rid of the wrinkles.”

NPCAs involving therapies considered complementary or alternative in some countries were described as routine care in other settings, e.g., Reiki and different forms of massage.

### Listening, Talking with, and Understanding

A wide variety of statements described dialogues aiming to understand and communicate desires, feelings, preferences, experiences, and interests and needs, particularly between professionals and the family, alone or as a unit including the dying person. It was less common that such NPCAs were expressed as directed only to the dying person, although when this was the case, staff described talking to both responsive and nonresponsive patients. The focus here is on verbal communication and interaction, with statements frequently using terms like “listening,” “asking,” “exploring,” “talking,” and “understanding.”

Statements described not only the content of verbal interaction, but also explained how one spoke and why, e.g., “speak with a low voice: often the patients say that high voices disturb them.” Staff also described intervening to have more frequent contact as death approached. Confirmation and legitimization about being in the last days of life, as well as words of comfort and solace, were topics for verbal communication in many NPCAs, although finding a comfortable balance could be described as challenging: “Not talking in front of the patient—is that good? That you go out to talk—want to talk in front of the patient! Not concealing information on one hand, creating peace and quiet for the dying person on the other hand.”

The content of conversations was not limited to the dying process, but included talking about daily life beyond sickness: “Help patient L with dinner. She was clearheaded and talked about the past and the new boyfriend of her daughter. Took the opportunity to chat somewhat longer, which made her cheer up.”

Even the time after death was addressed, often in relation to upcoming practical issues, but also in relation to celebration of life after the death of the involved person.

### Creating an Aesthetic, Safe, and Pleasing Environment

Many NPCAs were aimed at creating an aesthetic, safe, and pleasing environment for the dying person and his/her family. A wide repertoire of interventions to customize the environment was described and explained by individual preferences. In this category, we include efforts in both home and institutional settings to create beneficial physical, sensory, and personal environments. As examples of the latter, many personal care activities beyond basic hygiene needs, e.g., those to maintain skin integrity, were carried out. These included the use of perfumes, nail polish, hair care, and shaving, which were said to be chosen based on norms and habits from the person's previous daily life.

The physical environment was said to be adapted through use of color, textiles, different textures, and lighting, with a variety of strategies described that recognized the importance of sense of sight, e.g., “repositioning photographs around the bedside furniture into view” and “spread out dressing gown over white sheet, in order to get some contrast in color (face/sheet).” Music was used in a variety of situations, for both the sick person and his/her family, e.g., “same moaning again…music put on” and “turn on her favorite music.” NPCAs related to other aspects of the sensory environment—both physical and personal—were also common, e.g., regarding sound, “open the door, so life outside can be heard, birds, other people, the wind, children playing”; smell, “sprayed patient's favorite perfume on the sheet”; and touch, “scratch and massage the scalp, which I know the guest loves, comb and brush hair and put on the favorite hairclip.”

In addition to enhancing the environment, NPCAs also concerned efforts to diminish disturbing sensory experiences, as with the use of music above, and particularly related to odors around a dying person's deteriorating body (see also Figure 2): “Empty the wastepaper basket often, so that the room doesn't smell badly.” Creating an orderly, tidy, and clean environment was often described as involving the removal of things, both before and after death.

### Organizing, Planning, and Evaluating

NPCAs categorized as related to organizing, planning, and evaluating could involve direct patient and family contact, but also have “backstage” [19] components not witnessed by the recipients of care, including the planning and evaluation of the NPCAs. Activities that take place backstage include the following: completing paperwork such as death certificates or care plans, contacting other professionals and obtaining resources needed to optimize care, arranging logistics on a care unit, evaluating the need for medical tests or interventions, initiating and following end-of-life care pathways, and ensuring the availability and functionality of tools and technologies for comfort and care of the patient and family: “Arranging removal of hospital bed and wheelchair from house (for an inpatient) so family doesn't have to see this at home on their return once Dad dies.” As evident in this statement, such NPCAs continued even after the person had died.

Another important aspect of activities categorized here was fostering the involvement of the dying person and/or those close to the dying person in caregiving activities. This included determination of the extent to which this was desirable and/or beneficial for those involved, and was described in relation to both organizational issues and personal care: “For moving from one place to another (shifting places) or getting undressed: let the family members help if it is what the patient wishes.” The organization and reorganization of care was described as being carried out with respect for the patient's and family's own rhythms.

Evaluations of ongoing care, e.g., “ask ‘can I call tomorrow to hear how things are?’ especially if it feels uneasy,” as well as retrospective evaluations aimed at understanding family members' experiences of care (see Table 1) were both described. Staff consultations with one another were also mentioned as a form of evaluation in statements: “Do we [different staff members at the same unit] do things very differently? No, not so differently, we check with each other about what is said to the patient and significant others.”

### Observing and Assessing

Observations and assessments were notably often described as being carried out simultaneously with other interventions: “Some discomfort noticed, restlessness; comfort words given, holding her hands for a minute, CD player brought to her room.” Such NPCAs were directed towards family members and staff, as well, not only towards the dying person.

Verbs commonly used in NPCAs categorized here include “inspection,” “observation,” “surveillance,” “checking,” and “assessing,” carried out in relation to both responsive and nonresponsive dying patients. A particular form of assessment was related to diagnosing impending death: “Check the circulation of a dying patient with the aim of understanding where in the death process the patient is. Check the pulse, if hands and feet are warm. If the patient's lips are blue. Bluish or purple coloring of arms/legs or elsewhere. Look at the skin color, white around the nose and mouth.”

Visual observation and assessments seemed to occur more or less continually. Assessments were also said to occur through “talking with,” “exploring,” and “trying to understand” the needs and concerns of the dying person and family, as well as which positive factors could facilitate their comfort and well-being. Assessment of staff needs and well-being could include activities such as assessing group dynamics or professional identification with patient and family.

### Being Present and Enabling the Presence of Others

This category is comprised of NPCAs that are not reliant on verbal communication, but offer another form for communication. They often involved a staff person being in proximity to a dying person or his/her family. This was said to be done by sharing emotions or silences, often in an effort to calm through just sitting, with or without touching, but also through singing, reading aloud, or praying. NPCAs were also directed to facilitating the presence of others. This often referred to people close to the dying person directly or by proxy through messages from those unable to be at the bedside, but also included facilitating the presence of staff members, volunteers, and even pets. Presence was described as increasingly important close to death, as a means of easing situations for the dying person and families in difficult moments. Staff presence also offered a form of respite for family members. Presence without other intervention was said to sometimes demand courage on the part of the staff member, e.g., “dare to be silent with the dying person,” whereas on other occasions it involved silently witnessing celebrations of life during dying. Even here, abstaining from action was a purposeful form of caregiving: “Couple was cheering with champagne and music while one of them was dying: just stay there without speaking.”

### Performing Rituals Surrounding Death and Dying

The term “ritual” is used here in its broadest sense to include “social action in which a group's values and identity are publicly demonstrated or enacted…within the context of a specific occasion or event” [20]. As seen in Figure 1, much of the focus in this category is again on activities directed to families and staff rather than to the dying person alone. When the dying person was the focus, NPCAs were often related to religious rituals; other activities were formulated in general terms to describe an assessment of or effort to fulfill existential needs close to death. Religious and/or spiritual rituals, e.g., prayer, last rites, and funeral preparation and participation, took place in all countries despite their varied religious traditions and degrees of secularization.

A range of other more subtle rituals of legal and existential character are also included in this category, as are professional rituals around death and dying. Legal rituals included signing death certificates and providing routine advice to families about matters to be dealt with around a death, e.g., “talk about legal funeral papers with family.” Other rituals described in the process of preparing for death and taking leave of the person, both when dying and after death, might be viewed as existential in nature, e.g., “Understand the expectations and the wishes of the family (friends, relatives, intimates) about the care to the body after death. For example, let the wife lie on the bed next to her dead husband and hold him. The same for the children.”

As evident here, many types of rituals were described as having a high degree of flexibility rather than being entirely predetermined; there was a clear expression of the need to modify rituals in accordance with the preferences of the dying person and his/her family.

There were a notable number of both subtle and explicit professional rituals described in caring for the dying and deceased person, as well as for family members prior to, during, and after a death. Both professional staff and volunteers could describe changing their behaviors as death drew closer and peacefulness became more prioritized (see Table 1).

### Guiding and Facilitating

NPCAs categorized as guiding and facilitating include those described as intended to provide support in a compassionate manner, including practical support. These NPCAs contain a wide range of action verbs, e.g., “confirming,” “encouraging,” “facilitating,” “justifying,” “offering,” “reassuring,” “satisfying,” “sharing,” “showing possibilities,” “stimulating,” and “supporting.” These verbs indicate the staff member's intention to share his/her knowledge and experience to ease the situation for the dying person and family, with an implicit ideology of PC provision expressed. These NPCAs did not always describe verbal communication, sometimes instead presenting an effort to achieve a particular result without detailed description of how this was accomplished, e.g., “give new meaning to the word ‘hope’” and “find sources of pleasure.” Other descriptions are more pragmatic, e.g., “justify the crying of the patient when his intimates try to stop it,” and “helping press the right buttons on the cell phone.”

### Imparting Oral and Written Information and Advice

In contrast to the category “guiding and facilitating,” the focus here is on imparting information and advice, generally verbally. Verbs such as “explaining,” “advising,” “informing,” “training,” and “teaching” dominate. Written information about the dying process, support groups, and other resources were also provided for family members. Again, it is notable that, as seen in Figure 1, this is the NPCA category most rarely directed to the dying person alone according to the statements. When directed to the dying person, information was said to be conveyed regardless of level of consciousness (see Table 1).

The content of these NPCAs often related to explanations about changes that are occurring or to be expected as the dying process progresses, e.g., “explain to family about death rattles and other symptoms,” as well as teaching family members how to perform particular caregiving tasks. Information exchange among staff about changes in the condition or needs of the dying person is also included here (see Table 1).

## Discussion

In this article, we explore NPCAs during the last days of life, based on empirical data generated from 16 facilities in nine countries. We found that the multiple dimensions of PC agreed upon in theory are integrated in caregiving practice for the dying individual and his/her family. This integration might be understood metaphorically through how different threads—i.e., the different dimensions of physical, psychological, social, spiritual, and existential care expressed through NPCAs—are woven together into a complex tapestry. Whereas in theory these dimensions—threads—are often seen and discussed separately, in practice they are impossible to unravel, without losing the complexity and subtleties of the tapestry—i.e., how separate activities and dimensions are interwoven into PC practice. In these data, an underlying feature of the pattern of PC practice appears to be an effort to provide personalized and compassionate end-of-life care by maintaining and supporting links with the individual's everyday life.

A substantial portion of the NPCAs reported here related to bodily care and contact with both patients and family members, with refraining from carrying out bodily care also described as a purposeful part of care provision. However, this does not mean that the staff member did not provide other forms of care, e.g., often remaining present with the dying person. Communication was described in a variety of forms, with information and advice—directed more to family than to patients—at one end of a continuum, and communicating through nonverbal presence and bodily contact—mostly with patients—at the other. Rituals surrounding death and dying were not only related to spiritual/religious issues, but also included more subtle existential, legal, and professional rituals. An unexpected and hitherto little researched area of focus was on creating an aesthetic, safe, and pleasing environment in PC, both at home and in institutional settings (see also [21],[22]). Reflecting about caregiving also appears to be an activity intrinsic to care in the last days of life, seeming to function in part to maintain moral and/or ethical balance in efforts to achieve a “good death.” We also found it notable that in many statements, it was difficult to discern whether the person receiving care was still alive or deceased; death appeared to be conceptualized as a process rather than an occurrence at a fixed point in time.

We interpret many of the reported NPCAs as aiming to promote well-being and comfort through maintaining connections to the individual's everyday life. Connections to everyday life were fostered through a wide variety of activities, such as adapting the environment to accommodate the person's earlier life and habits (e.g., playing favorite music, using own creams, placement of photographs). This adaptation is accomplished by using knowledge about and respect for the person as an individual with a life history lived in a particular context, i.e., the person is not viewed only as a dying patient. These data thus add new substance to the commonly used terms patient- or person-centered care [23],[24] by illustrating their application in practice.

The importance of what is often described as “small talk” becomes evident here as a basis for care provision. It is notable that when activities refer to talking about subjects that are not specifically disease- or problem-focused, they tended to be trivialized by use of diminutive terms, e.g., “chatting.” Such terminology does not acknowledge these contacts as central in providing an understanding needed to transfer general principles into the situation-specific knowledge underlying the provision of patient/person-centered care. This can be seen as parallel to a societal tendency to view technological and medical treatments as having higher status than non-pharmacological caregiving [25]. Similarly, bodily care for the dying person is often conceptualized as “basic” care [26]. Based on these data, we argue that providing for fundamental human needs close to death is instead complex and sophisticated (see also [27]), as exemplified with mouth care: such care appears to be based on a series of decisions not only about what is to be done or not done, but also how, why, when, and for whom it is done. It is necessary to better distinguish nuances in non-pharmacological caregiving in order to acknowledge, respect, and further develop this type of care.

Müller and Cox Dzurec [28], among others, support this as they emphasize how thinking and actions are directly correlated to the language used in conceptualizing caregiving phenomena. We have directly experienced the challenges in and importance of name-giving when working with this dataset. Statements were most often written in lived and experiential, rather than abstract, language. The process of categorization involved an abstraction of these data, but we found that we lacked language with which to express the integration of activities found in these composite descriptions, turning to metaphor instead. Fragmentation between mind and body has previously been discussed elsewhere (see e.g., [29],[30]). The concept of “total pain” focuses on the importance of including physical, psychological, social, spiritual, and existential aspects [4],[5]. Despite this, several years ago Corner and Dunlop [31] pointed out that these aspects were often conceptualized as separate entities, and treated as such; this is still the case, for example, in many of the major textbooks in palliative medicine (see e.g., [7]–[9]), and perhaps a necessity in educational situations. However, in these data we note how staff document further and more seamlessly integrating these separate entities in caregiving, although more nuanced terms of description for this integration are still lacking in Western languages.

In these data, staff described different forms of interaction with individual patients and families, with different degrees of dependency on verbal communication. The four categories—listening, talking with, and understanding; being present and enabling the presence of others; guiding and facilitating; and imparting oral and written information and advice—are closely linked in their relationship to communication. However, one risk in labeling them as such is that nuances in communication and the importance of communicating through other means, e.g., via bodily contact, would not be recognized, thereby further cementing the division between bodily care and psychosocial care that dominates the existing literature.

A number of factors should be kept in mind when considering these data. Despite instructions to document each different activity once for each patient, this was not consistently the case, although we have compensated for this in the analysis. The variation in the manner in which free-listing has been used at different units prohibits us from drawing any conclusions beyond those intended on diversity in NPCAs, although it is likely that these data underrepresent the diversity of activities actually occurring in clinical practice. The generated data were sent to us in the original language with an English translation, which means that nuances may have been lost in translation. In most cases, we are also unaware of which statements were derived from group discussions versus the follow-up writing of the documentation; differences in expressions and their tenor may in part be related to the form for documentation as well as the translation process.

It should be recognized that these data are not generalizable as to frequency of occurrence beyond this dataset. As our intention was to investigate diversity, correlational conclusions should not be drawn with regard to profession or country; we note that teams are constructed differently, with similar functions carried out by different professional groups in different contexts. Some positions are notably specific to particular facilities, e.g., those related to staff support, but have contributed to an understanding of diversity in caregiving activities, in accordance with the aim of this study.

The relationship between talking or writing about what one does, and what is actually done should also be considered here; based on these data, we are unable to evaluate the effectiveness or benefit of the NPCAs described. There is a risk that an idealized picture of end-of-life care in general, and of non-pharmacological caregiving in particular, was documented. This is especially apparent in the use of positively laden terms like “support,” “understanding,” and “confirming,” which clarify an intention, but are formulated as being a result of an intervention in many statements. This highlights the need for innovative approaches to complement these staff-derived data by evaluating the outcomes of NPCAs for the recipients of care, without the data being colored by the intentions of the caregivers. Participant observation could complement this dataset and generate new findings and questions of relevance, as well as help pinpoint relevant outcome variables for further research.

Despite these caveats, these empirical data address existing knowledge gaps that need to be filled to secure a stable evidence base for improving non-pharmacological caregiving in the last days of life, and a number of areas needing further investigation were noted. For example, there is little empirical research regarding the sensory environment for the dying, although numerous activities to optimize environmental features such as sights, smells, sounds, and general atmosphere were described; some of these are recognized in newly published guidelines for quality end-of-life environments [32]. Again, there is a notable lack of adequate terminology to reflect the complexity of NPCAs involving the body at the end of life. Literature needs to describe care for the body with greater sophistication, doing justice to the variety and intricacies in the descriptions reported in this dataset. Allowing a greater level of detail would make possible an appreciation of the patterns, nuances, and complexity present in the tapestry of non-pharmacological care provision during the last days of life, with potential benefit for clinical practice, teaching, and research.

## Abbreviations

NPCA: non-pharmacological caregiving activity
PC: palliative care
